# Biological Indicators of Cardiovascular Health by Foster Care History in Adults

**DOI:** 10.1016/j.amepre.2025.108097

**Published:** 2025-09-12

**Authors:** Darlynn M. Rojo-Wissar, Sean R. Womack, Tomas Baka, Adam P. Spira, Ryan D. Davidson, Eric S. Zhou, Candice A. Alfano, Chandra L. Jackson, Michael A. Grandner, Stephanie H. Parade

**Affiliations:** 1 Department of Psychiatry and Human Behavior, Alpert Medical School of Brown University, Providence, Rhode Island; 2 Bradley/Hasbro Children’s Research Center, E.P. Bradley Hospital, East Providence, Rhode Island; 3 Center of Biomedical Research Excellence for Stress, Trauma, and Resilience, The Miriam Hospital, Providence, Rhode Island; 4 Institute of Pathophysiology, Faculty of Medicine, Comenius University, Bratislava, Slovakia; 5 Department of Mental Health, Johns Hopkins Bloomberg School of Public Health, Baltimore, Maryland; 6 Department of Psychiatry and Behavioral Sciences, Johns Hopkins School of Medicine, Baltimore, Maryland; 7 Center on Aging and Health, Johns Hopkins University, Baltimore, Maryland; 8 Boston Children’s Hospital, Boston, Massachusetts; 9 Harvard Medical School, Boston, Massachusetts; 10 Department of Psychology, University of Houston, Houston, Texas; 11 Epidemiology Branch, National Institute of Environmental Health Sciences, NIH, HHS, Research Triangle Park, North Carolina; 12 Intramural Program, National Institute on Minority Health and Health Disparities, NIH, HHS, Bethesda, Maryland; 13 Department of Psychiatry, University of Arizona College of Medicine, Tucson, Arizona

## Abstract

**Introduction:**

Childhood adversity contributes to adult cardiovascular health disparities, but the cardiovascular health of foster care alumni, who experience unique compounded stressors such as attachment disruption and environmental upheaval, is understudied. In this study, biological cardiovascular health indicators were described among U.S. adults with and without a foster care placement history.

**Methods:**

Cross-sectional data from 4,625 adults (representing 17,226,361 U.S. adults) approaching and in early midlife (2016–2018) from The National Longitudinal Study of Adolescent to Adult Health were used. Biological indicators of cardiovascular health included BMI, blood lipids, blood glucose, and blood pressure, which were each scored on a standardized scale of 0–100, with higher scores indicating better cardiovascular health. An unweighted average of these standardized scores was also computed. Incorporating sampling weights, nationally representative estimates of cardiovascular health by foster care history were generated in 2024–2025.

**Results:**

There were 113 participants who reported a foster care placement history (1.8% [weighted]), representing 313,604 adults. Foster care alumni had poorer overall cardiovascular health scores (mean=61.8, 95% CI=56.6, 66.9) and blood pressure health scores (mean=48.7 [indicates hypertension], 95% CI=39.8, 57.5) than those without a foster care history (cardiovascular health: mean=70.2, 95% CI=69.1, 71.4; blood pressure: mean=61.6, 95% CI=59.9, 63.2). Notably, common protective factors (e.g., female sex, higher income) did not mitigate cardiovascular health risk in the foster care group.

**Conclusions:**

Childhood foster care placement is associated with poorer cardiovascular health, particularly hypertension, even in groups generally at lower risk. Research and clinical initiatives are needed to better understand and address cardiovascular health inequities and promote cardiovascular wellness in this population.

## INTRODUCTION

A recent American Heart Association (AHA) paper identifies a lack of attention to adversity in cardiovascular medicine research and practice as a major source of health disparities and emphasizes the need to invest in adversity-related science to improve cardiovascular health (CVH) nationally.^1^ The importance of childhood adversity, in particular, has been highlighted, defined as “subjectively perceived threats to the safety or security of the child’s bodily integrity, family, or social structures.”^2^ Although foster care (FC) is not directly mentioned in this scientific statement, it represents a population of over 400,000 children in the U.S.^3^ who face significant adversity. Compounded experiences of abuse, neglect, caregiver loss, environmental upheaval, and placement instability make this group a critical target for CVH research and intervention work. Indeed, adults with a FC placement history are 50%–100% more likely to develop cardiovascular disease (CVD).^4,5^ They are also more likely to experience CVD-related hospitalization and death than those who did not experience FC.^6^

Increased risk for CVD among those with a history of FC may occur through biological embedding of stress, which can result in substantial allostatic load, leading to dysregulation of metabolism, lipids, glucose, adipose tissue, and an increased risk of hypertension.^1^ Indeed, BMI, blood lipids, blood glucose, and blood pressure are key biological indicators of CVH^7^ and are not only associated with the development of CVD but also with a variety of other poor health outcomes,^8,9^ including mortality.^10^ Unfortunately, there is little research evaluating these key biological indicators of both the biological embedding of stress and of CVH risk in the FC population. The sparse work that does exist has largely neglected to examine these biological indicators objectively or as a reflection of health on a continuum and has instead often relied on self-reported diagnoses.^11–13^ Previous work is also limited by the grouping of people from several racial minority groups into a single other category, potentially obscuring important group differences.^11–13^ Addressing these knowledge gaps, especially in adults approaching and in early midlife, a developmental period directly prior to the average CVD onset,^14^ is vital for improving CVH and preventing CVD in individuals with a history of FC.

Data from the nationally representative National Longitudinal Study of Adolescent to Adult Health (Add Health) was used to describe multidimensional CVH in adults with and without a FC history. This included scores for objectively measured BMI, blood lipids (non–high-density lipoprotein [HDL] cholesterol), blood glucose, blood pressure, and an overall score across these domains. In the context of the limited available literature, it was hypothesized that these continuous biological indicators of CVH would generally be poorer among those with a history of FC placement than among those without a FC placement history. The prevalence of these biological indicators of CVH by relevant psychosocial factors^7,15,16^ was also explored to identify patterns and generate hypotheses for future research.

## METHODS

### Study Sample

The Add Health, which began recruitment of participants in Grades 7–12 during the 1994–1995 school year, is one of the largest and most comprehensive nationally representative longitudinal studies of American adolescents to date (N=20,745).^17^ Sampling for Add Health consisted of a school-based sampling design where high schools were selected through stratified sampling on the basis of factors such as school size, type, and census region.^18^

For the current analysis, data were used from Wave V (2016–2018), in which participants were aged 33–43 years (mean=37.13 [SE=0.12]), and relevant data on the biological indicators of CVH were collected to track the emergence of chronic disease as participants advanced in age. Anthropometric, cardiovascular, metabolic, and inflammatory measures were obtained through examinations and blood draws/tests. Other data were collected using a mixed-mode survey, with questionnaires administered through interviewer during home visits, by telephone, through the web, or by mail.^17^ There were 5,381 participants who completed the Wave V home examination and had a biomarker sample weight. The analytic sample consisted of 4,625 of these participants (Table 1) who also had a cluster variable to identify groups of students within the same schools, a poststratification variable to identify census region from when they were first recruited, and who had data on FC history (assessed in Wave III). This secondary analysis of deidentified data was deemed exempt from oversight by Brown University’s IRB.

### Measures

Regarding the biological indicators of CVH, Add Health field examiners measured participant height and weight and blood pressure, collected health history and medication information, and collected venous blood samples from participants, which were assayed for lipid and glucose concentrations. These data were used to create standardized scores for each biological indicator (BMI, blood pressure, blood glucose, blood lipids [non-HDL cholesterol]) on a scale of 0 to 100 using AHA criteria. Scores on the 4 indicators were also averaged to create an overall CVH score, with higher scores indicating better CVH across all biological indicators. AHA scoring criteria were applied to these stress-related biological indicators of CVH (the 4 health factors in Life’s Essential 8)^7^ owing to its widespread adoption as a standardized way to measure individual components of CVH and CVH overall. Its large-scale use for monitoring population CVH and intervention effectiveness facilitates greater comparability of findings across studies, particularly in population-based research. The AHA scoring criteria also allow for comparability across the individual biomarkers by harmonizing the continuous measures into a standardized scale and incorporating key clinical considerations—such as disease status and medication use—into its scoring algorithm. The standardized scores are easy to interpret, and the scoring indexes have been widely validated in prior studies. For example, the 4 health factors are associated with the development of CVD and a variety of other outcomes, including dementia,^19^ advanced biological aging,^8^ depression,^9^ and both CVD-specific mortality and all-cause mortality.^10^ Although there is some evidence that overall CVH scores can be accurately estimated in situations where not all 8 metrics are available,^20^ the overall CVH score in this paper may be most representative of biological indicators of CVH. Additional measurement and scoring information are available in Appendix Tables 1 and 2 (available online).^7^

FC history status was retrospectively assessed with the question, *Did you ever live in a foster home?* Maltreatment in foster or adoptive care and the number of placements were also reported. Age was computed by subtracting participants’ birthdates from the date of their Wave V visit. Participants reported their biological sex assigned at birth (male, female), their race and ethnicity (categorized as non-Hispanic/Latino White, Black, American Indian or Alaskan Native, Asian, Pacific Islander, other race, multiracial, or Hispanic/Latino), and marital status (married, widowed, divorced, separated, never married). Poverty index scores (low income ≤1.30, low middle income of 1.31–1.85, middle income of 1.86–3.50, and high income >3.50) were computed using household annual income and size using federal poverty guidelines.^15^ The 5 items from the negative affect subscale of the Center for Epidemiologic Studies-Depression scale^21^ were used to assess total depressive symptoms. A dichotomous variable indicating those in the bottom and top 50% of scores (0/1) was used in analyses to approximate relative symptom burden in lieu of an established clinical cutoff.

### Statistical Analysis

To generate unbiased estimates and retain national representativeness, design-based analyses incorporating sampling weights, school-level clustering, and regional poststratification were applied in STATA, Version 16.0 (StataCorp, College Station, TX). First, the sample was described; then, mean CVH indicators by FC history across contextual characteristics were estimated. Differences were considered meaningful if 95% CIs did not overlap. Finally, to further contextualize these findings, supplemental analyses were conducted among those with a FC history, examining mean CVH scores by maltreatment in foster or adoptive care and multiple placements. All available data were used; thus, sample sizes vary slightly owing to mostly minor missingness (Appendix Table 3, available online).

## RESULTS

There were 113 participants (weighted *n*=313,604) with a FC placement history and 4,512 participants (weighted *n*=16,912,757) without a FC placement history (Table 1). Those with a FC history had a lower proportion of participants who were White or Hispanic/Latino of all races and a larger proportion who were Black and multiracial than those without a FC history. Those with a FC history also had a lower proportion of married participants; a higher proportion of divorced, separated, or never-married participants; and a greater proportion of participants in the lower ends of the poverty index and in the top 50% of depressive symptom scores.

**Figure 1.**
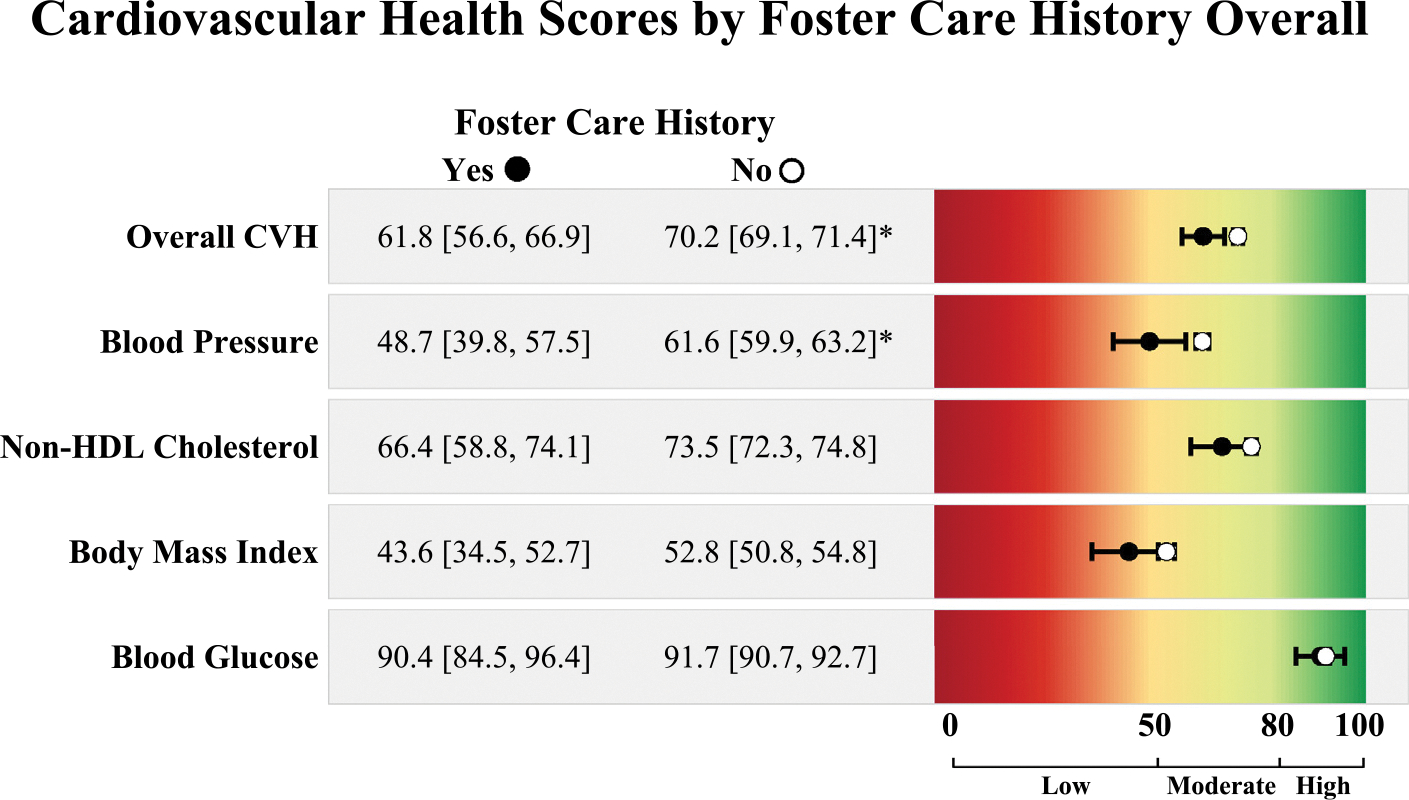
Biological indicators of CVH scores by foster care placement history. *Note*: Point estimates and 95% CIs are in black for those with a foster care history and in white for those without a foster care history. Mean scores are considered different by foster care history if 95% CIs do not overlap and are indicated by asterisks. Scores range from 0 to 100, with scores of 80–100 (green) considered high CVH, 50–79 (yellow) considered moderate CVH, and scores of 0–49 (red) considered low CVH. CVH, cardiovascular health.

Scores for the CVH outcomes are reported as mean (95% CI). Participants with a FC history had lower overall CVH scores (61.8 [56.6, 66.9]) than those without a FC history (70.2 [69.1, 71.4]) (Figure 1). For the individual biological indicators, blood pressure scores were significantly lower in adults with a FC placement history (48.7 [39.8, 57.5]; scores <50 indicative of hypertension) than in those without (61.6 [59.9, 63.2]). There were no meaningful differences in non-HDL cholesterol, BMI, or blood glucose scores by FC placement history, although non-HDL cholesterol and BMI were trending toward poorer health in those with a FC history. Finally, only 14.7% of those with a FC history had ideal CVH (i.e., no biological indicators of CVH in the low range [≤50]), compared with 27.4% without (*p*=0.049).

**Figure 2.**
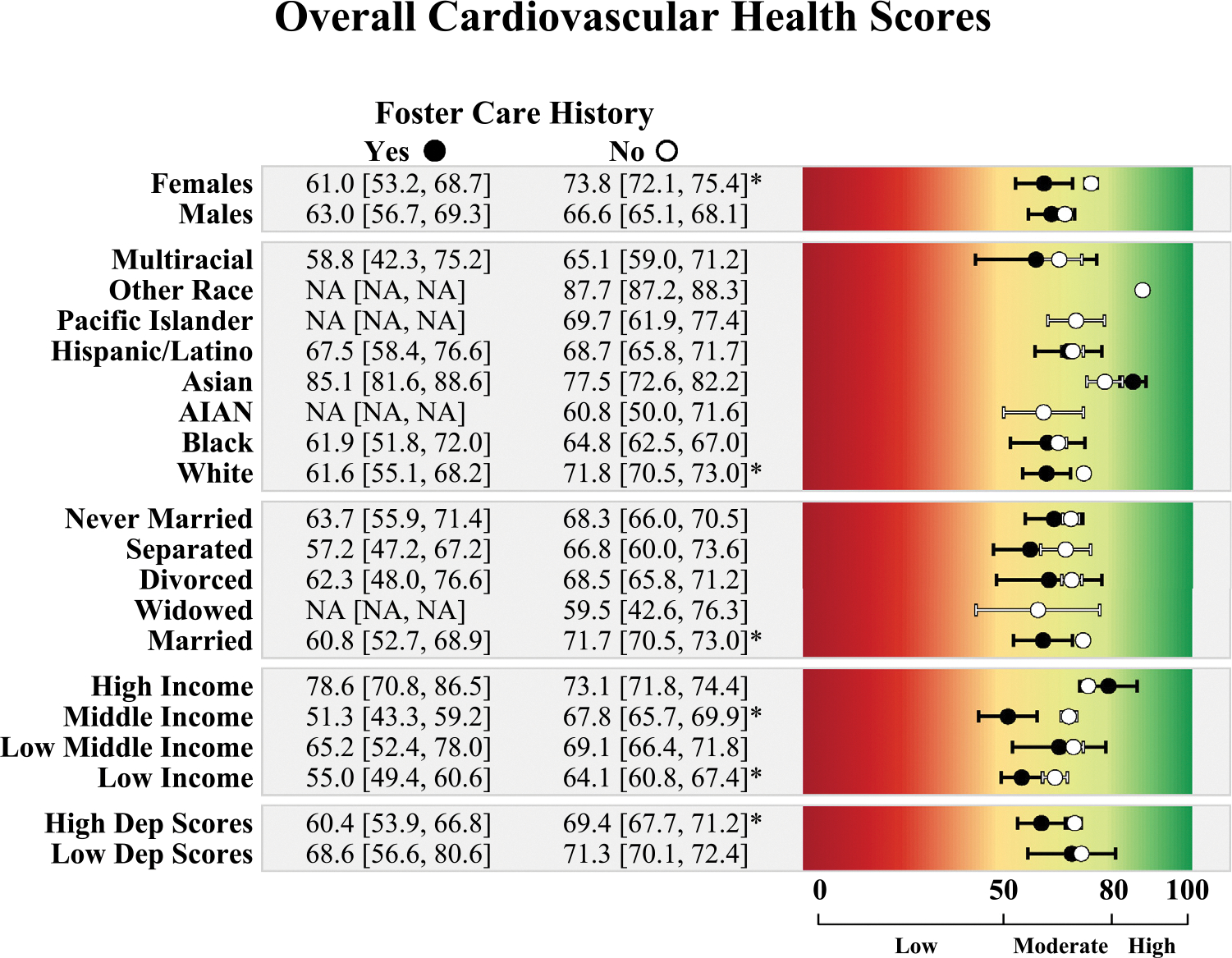
Overall CVH scores by contextual factors among those with and without a foster care placement history. *Note*: High dep scores=top 50% depressive symptom scores, and low dep scores=bottom 50% depressive symptom scores. Point estimates and 95% CIs are in black for those with a foster care history and in white for those without a foster care history. Mean scores are considered different by foster care history if 95% CIs do not overlap and are indicated by asterisks. Groups with 2 few ns to obtain estimates are labeled as NA. Scores range from 0 to 100, with scores of 80–100 (green) considered high CVH, 50–79 (yellow) considered moderate CVH, and scores of 0–49 (red) considered low CVH. AIAN, American Indian or Alaska Native; CVH, cardiovascular health; NA, not applicable.

**Figure 3.**
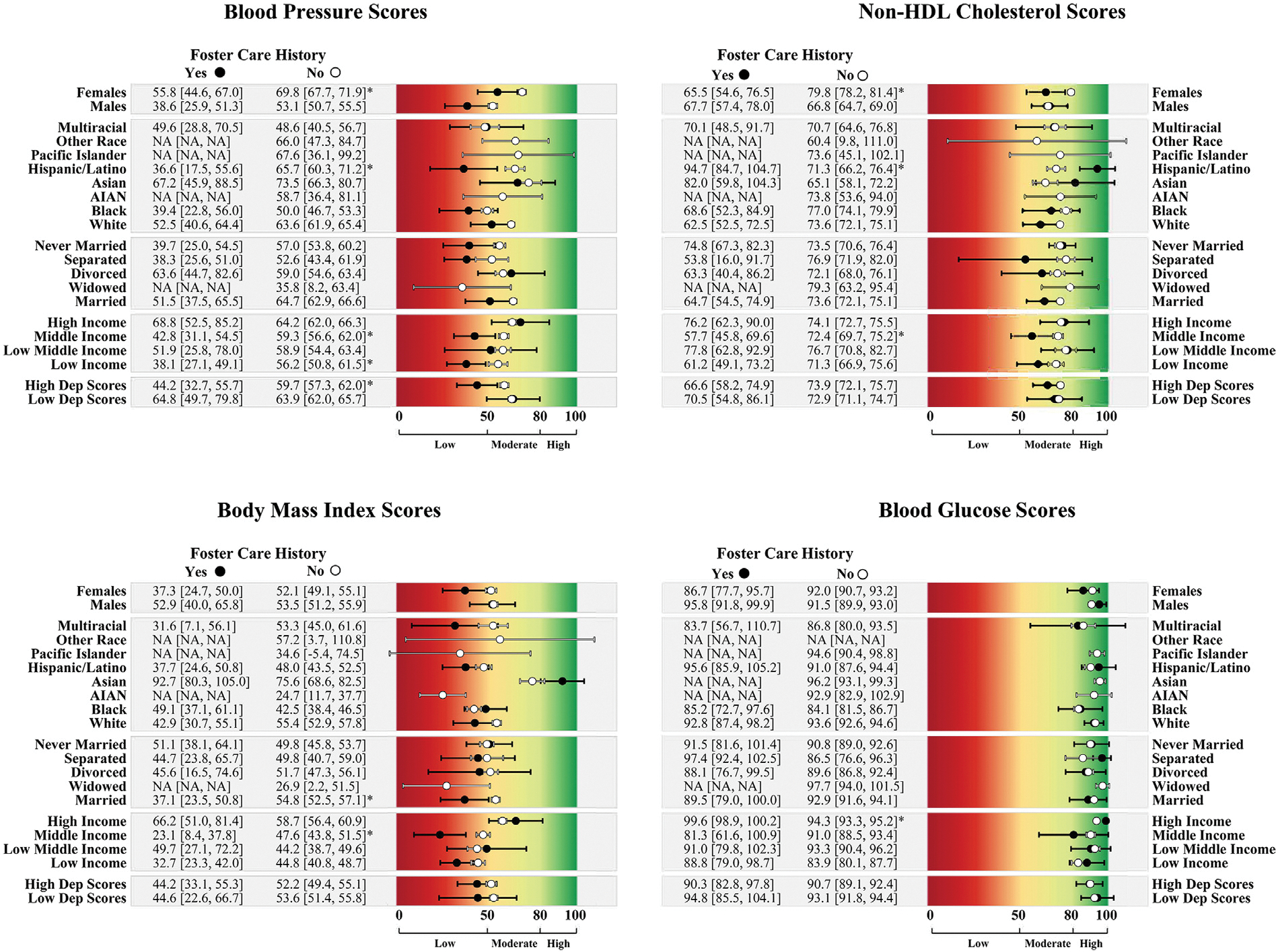
CVH scores for the individual biological indicators by contextual factors among those with and without a foster care placement history. *Note*: High dep scores=top 50% depressive symptom scores, and low dep scores=bottom 50% depressive symptom scores. Point estimates and 95% CIs are in black for those with a foster care history and in white for those without a foster care history. Mean scores are considered different by foster care history if 95% CIs do not overlap and are indicated by asterisks. Groups with 2 few ns to obtain estimates are labeled as NA. Scores range from 0 to 100, with scores of 80–100 (green) considered high CVH, 50–79 (yellow) considered moderate CVH, and scores of 0–49 (red) considered low CVH. AIAN, American Indian or Alaska Native; CVH, cardiovascular health; NA, not applicable.

Stratified CVH scores by FC history and contextual factors are reported in Figures 2 and 3. Overall, CVH scores were lower among adults who were female, White, married, from low- and middle-income brackets, and in the top 50^th^ percentile of depressive symptom scores for those with a FC history than for those without.

Among individuals who were female, Hispanic/Latino, low-income, middle-income, and in the top 50^th^ percentile of depressive symptom scores, those who had a FC history had lower blood pressure health scores than those without a FC history. For blood lipids health, those with a FC history had a lower score than those without a FC history if they were female or in the middle-income bracket. Among Hispanic or Latino individuals, the opposite pattern emerged, where those with a FC history had better blood lipid health scores than those without a FC history.

BMI health scores were significantly lower in married adults with a FC history than in married adults without. Among middle-income adults, those with a FC history had significantly lower BMI health scores than those without. Counterintuitively, among those in the high-income range, those with a FC history had higher blood glucose health, although mean scores for both groups were in the high blood glucose health range.

CVH scores were generally similar regardless of maltreatment history in foster or adoptive care or number of FC placements. However, most individuals with a maltreatment history had notably lower blood pressure scores, all of which were within the low health range (Appendix Figures 1 and 2, available online).

## DISCUSSION

Using data from a nationally representative sample of American adults approaching and in early midlife, reporting childhood FC involvement was associated with a greater risk for poorer CVH in this study. Findings also suggest that CVH disparities may be exacerbated when intersected with other relevant contextual factors (e.g., sex, race and ethnicity, SES). Current AHA guidelines recommend integrating psychosocial stressors and other social determinants of health in cardiovascular risk assessment and in the implementation of treatment recommendations for the prevention of CVD.^22^ Findings from this study support the consideration of FC history as a social determinant of CVD,^23^ which could improve cardiovascular risk assessment and open avenues for early interventions focused on alleviating the effects of exposure to adversity on long-term CVH. Investment in CVH promotion efforts in this population is critical for increasing health equity in this group at a disparately higher risk for CVD.

Identifying CVH risk factors prior to midlife is crucial. Although the prevalence of CVD is relatively low during this period,^14^ health factors that pose a risk for CVD (e.g., elevated blood pressure, obesity) are often present decades before disease onset.^24,25^ Adults with a FC placement history had meaningfully lower blood pressure health scores and CVH scores overall, implicating FC history as a potential risk factor for poorer CVH in early midlife. In the study sample, which is representative of over 17 million American adults aged 33–43 years, the mean blood pressure score for adults with a FC placement history fell in the hypertensive range. This finding is particularly concerning given that several studies have demonstrated that elevated blood pressure in young adulthood and early midlife increases the risk for CVD and related mortality in later life above and beyond traditional cardiovascular risk factors.^26–28^ This suggests that FC placement history represents an important social factor in the developmental course of hypertension and CVD.

Although scores on other biological indicators of CVH (i.e., lipids, glucose, BMI) were not significantly different for individuals who experienced FC relative to those who did not, blood lipid (non-HDL cholesterol) and BMI health scores were trending toward being significantly poorer among those with a FC history. Blood pressure may be more reactive and responsive to stressors experienced by children who are removed from their homes than to other biological indicators of CVH through its connection with the hypothalamic–pituitary–adrenal axis and sympathetic nervous system.^29,30^ FC-related stressors may affect other biological indicators of CVH more slowly over time and less directly. Future research will elucidate the part of the life course when trends toward non-HDL cholesterol and BMI differences by FC history become significant. Contrary to non-HDL cholesterol and BMI, blood glucose health scores were very similar by FC history. This could be explained by the homeostatic counterregulation that glucose levels are subject to, particularly with regard to insulin.^31^ The authors posit that among people in this age range, the body may still be compensating for stress-induced dysregulation through insulin’s modulatory effects, maintaining normal levels of blood glucose despite underlying physiological strain. Differences in blood glucose levels may be observable at much older ages than the other biological indicators of CVH.

Subgroup analyses by sex, race/ethnicity, income, marital status, and depressive symptoms revealed nuanced patterns in CVH differences by FC history. In brief, among those without a FC history, males had poorer CVH than females across most biological indicators of CVH, but this sex difference was not seen among those with a FC history—suggesting that FC may negate the protective effect of being female. Disparities in overall CVH by FC history were only evident among White adults, possibly owing to the cumulative stress experienced by racially minoritized groups. Among Hispanic adults, those with a FC history had poorer blood pressure health but better non-HDL cholesterol health than those without, which should be examined further in future research. Differences by income and marital status suggest that FC may undermine the protective effects of higher income and marriage. Among adults with high depressive symptoms, those with a FC history had poorer blood pressure health. When examining differences in CVH by traumatic FC experiences, it appeared that blood pressure health may be further negatively affected among people with a FC history if they also experienced maltreatment in foster or adoptive care. These hypothesis-generating findings are discussed further in the Appendix (available online) but should be interpreted with caution owing to small sample sizes in some subgroups. Future studies should examine whether findings across subgroups replicate in larger, more diverse samples of people with a history of FC placement. However, findings highlight a likely need for comprehensive support across multiple domains (e.g., relationship counseling, financial resources) for people who experience FC to ensure their well-being and successful development into adulthood.

### Limitations

This study has limitations. First, FC placement history was assessed using a single self-report question, which may lead to measurement error related to recall bias, underreporting, or misclassification. For example, some people might not consider kinship placements to be FC, and some might not remember short-term placements. Second, important contextual information on placement history was absent, such as age of placement, re-entry into FC, duration of placement(s), and kinship versus nonrelative FC. These contextual factors should be examined in future studies and may be important in differentiating levels of risk and identifying potential protective factors associated with specific FC experiences. Third, CVH outcomes were limited to 1 time point, prohibiting the testing of developmental patterns of CVH, which should be examined in future studies to identify periods of risk and inform intervention timing. Despite the limitations, this study has several noteworthy strengths. First, the use of a large, diverse cohort study representative of over 17 million American adults is a strength. The study sample’s age range allowed to provide novel descriptions of biological indicators of CVH among adults approaching and in early midlife, prior to the typical age of CVD onset. Finally, measures of CVH were collected by trained phlebotomists during in-home assessments, which represents a methodologic advantage over studies that rely on self-reports of disease status.

## CONCLUSIONS

In summary, experiencing FC placement in childhood may be a substantial social risk factor for poorer CVH in adults approaching and in early midlife, particularly with regard to blood pressure. Furthermore, this study provides evidence that FC history contributes to CVH disparities among adults within sociodemographic groups that are generally considered to be protected against developing CVH problems, such as females, people who have moderate to high income, and those who are married. AHA guidelines recommend incorporating childhood adversity and lifetime social stressors into CVD risk assessments.^22^ In light of these findings, the authors suggest the inclusion of FC placement. Targeted CVH interventions may be necessary to address the unique needs of the FC population. Because FC placement occurs during childhood and often coincides with an increased level of service involvement, there is potential for early intervention. Future longitudinal research among children and adults with a FC placement history is needed to confirm these findings, determine the mechanisms linking placement to poorer CVH outcomes, and identify key points of intervention. These efforts are crucial for mitigating CVH disparities and promoting health equity and wellness in this group.

## Supplementary Material

Supplementary Materials

Supplemental materials associated with this article can be found in the online version at https://doi.org/10.1016/j.amepre.2025.108097.

## Figures and Tables

**Table 1.** Characteristics of U.S. Adults by FC Placement History, Weighted n (%)

		FC placement history	
Characteristics	Total sample (n=4,625), weighted to 17,226,361 adults	Yes (*n*=113) weighted to 313,604 (1.8%)	No (*n*=4,512) weighted to 16,912,757 (98.2%)

Age, years, mean (SE)	37.13 (.12)	37.16 (.31)	37.13 (.12)
Sex (female)	8,860,949 (51.4)	184,564 (51.3)	8,676,384 (58.9)
Race and ethnicity			
NH White	11,584,697 (67.4)	182,263 (58.1)	11,402,434 (67.6)
NH Black	2,592,980 (15.1)	73,968 (23.6)	2,519,012 (14.9)
NH AIAN	72,308 (0.42)	0 (0)	72,308 (0.43)
NH Asian	397,180 (2.3)	5,174 (1.6)	392,006 (2.3)
Hispanic or Latino	1,824,239 (10.6)	12,719 (4.1)	1,811,520 (10.7)
Pacific Islander	30,808 (0.18)	0 (0)	30,808 (0.18)
NH other race	37,767 (0.22)	208 (<1)	37,559 (0.22)
NH multiracial	638,327 (3.7)	39,272 (12.5)	599,055 (3.6)
Marital status			
Married	9,826,517 (57.2)	128,977 (41.1)	9,697,540 (57.5)
Widowed	52,334 (0.30)	0 (0)	52,334 (.31)
Divorced	2,227,111 (13.0)	55,078 (17.6)	2,172,033 (12.9)
Separated	593,306 (3.5)	26,011 (8.3)	567,296 (3.4)
Never married	4,495,358 (26.1)	103,539 (33.0)	4,391,819 (26.0)
Poverty index			
Low income	2,416,748 (15.5)	112,635 (40.1)	2,304,113 (15.0)
Low middle income	1,101,648 (7.1)	60,128 (21.9)	1,041,520 (6.8)
Middle income	3,698,119 (23.5)	62,325 (22.7)	3,605,794 (23.5)
High income	8,432,104 (54.0)	39,752 (14.5)	8,392,353 (54.7)
Depression			
CES-D score, mean (SE)	7.44 (.07)	9.53 (.50)	7.40 (.06)
Top 50% CES-D Scores	9,052,402 (53.4)	241,942 (79.0)	8,810,460 (53.0)

*Note*: One participant was missing data on age (no FC history group), 0 were missing data on sex, 13 on race and ethnicity (no FC group), 8 on marital status (no FC group), 426 on the poverty index (14 of these were from FC group), and 60 on depressive symptoms (3 from FC group).

AIAN, American Indian or Alaska Native; CES-D, Center for Epidemiologic Studies Depression Scale; FC, foster care; NH, non-Hispanic.

## References

[1] Westcott SK, Lewis TT, Albert MA. Tackling adversity and cardiovascular health: it is about time. Circulation. 2023;147(1):e1–e3. 10.1161/CIRCULATIONAHA.122.061763. 36576955

[2] Suglia SF, Koenen KC, Boynton-Jarrett R, Childhood and adolescent adversity and cardiometabolic outcomes: a scientific statement from the American Heart Association. Circulation. 2018;137(5):e15–e28. 10.1161/CIR.0000000000000536. 29254928 PMC7792566

[3] In foster care on the last day of FY. Children’s Bureau. https://cwoutcomes.acf.hhs.gov/cwodatasite/inCareSeptemberThirty/index. Updated 2022. Accessed July 25, 2022.

[4] Batty GD, Kivimäki M, Almquist YB, Cardiovascular disease events in adults with a history of state care in childhood: pooling of unpublished results from 9 cohort studies. medRxiv. Preprint. Online January 27, 2024. 10.1101/2024.01.26.24301814.

[5] Hjern A, Vinnerljung B, Brännström L. Cardiovascular disease and risk factors in individuals with a history of out-of-home care. Pediatrics. 2024;153(2):e2023063174. 10.1542/peds.2023-063174. 38263888

[6] Jackisch J, Almquist YB. Childhood adversity is associated with hospitalisations and survival following external causes and non-communicable diseases: a 46-year follow-up of a Stockholm birth cohort. J Epidemiol Community Health. 2023;77(4):209–215. 10.1136/JECH-2022-219851. 36737239 PMC10086507

[7] Lloyd-Jones DM, Allen NB, Anderson CAM, Life’s essential 8: updating and enhancing the American Heart Association’s construct of cardiovascular health: a presidential advisory from the American Heart Association. Circulation. 2022;146(5):e18–e43. 10.1161/CIR.0000000000001078. 35766027 PMC10503546

[8] Zhang R, Wu M, Zhang W, Association between life’s essential 8 and biological ageing among U.S. adults. J Transl Med. 2023;21(1):622. 10.1186/s12967-023-04495-8. 37710295 PMC10503107

[9] Zeng G, Lin Y, Lin J, He Y, Wei J. Association of cardiovascular health using Life’s Essential 8 with depression: findings from NHANES 2007–2018. Gen Hosp Psychiatry. 2024;87:60–67. 10.1016/j.genhosppsych.2024.01.011. 38306947

[10] Sun J, Li Y, Zhao M, Association of the American Heart Association’s new “Life’s Essential 8” with all-cause and cardiovascular disease-specific mortality: prospective cohort study. BMC Med. 2023;21(1):116. 10.1186/s12916-023-02824-8. 36978123 PMC10053736

[11] Ahrens KR, Garrison MM, Courtney ME. Health outcomes in young adults from foster care and economically diverse backgrounds. Pediatrics. 2014;134(6):1067–1074. 10.1542/PEDS.2014-1150. 25367543 PMC4243069

[12] Zlotnick C, Tam TW, Soman LA. Life course outcomes on mental and physical health: the impact of foster care on adulthood. Am J Public Health. 2012;102(3):534–540. 10.2105/AJPH.2011.300285. 22390519 PMC3487656

[13] Turney K, Wildeman C. Mental and physical health of children in foster care. Pediatrics. 2016;138(5):e20161118. 10.1542/peds.2016-1118. 27940775

[14] Yazdanyar A, Newman AB. The burden of cardiovascular disease in the elderly: morbidity, mortality, and costs. Clin Geriatr Med. 2009;25(4):563–577. 10.1016/j.cger.2009.07.007. 19944261 PMC2797320

[15] Lloyd-Jones DM, Ning H, Labarthe D, Status of cardiovascular health in U.S. adults and children using the American Heart Association’s new “Life’s essential 8” metrics: prevalence estimates from the National Health and Nutrition Examination Survey (NHANES), 2013 through 2018. Circulation. 2022;146(11):822–835. 10.1161/CIRCULATIONAHA.122.060911. 35766033

[16] Wong CW, Kwok CS, Narain A, Marital status and risk of cardiovascular diseases: a systematic review and meta-analysis. Heart. 2018;104(23):1937–1948. 10.1136/heartjnl-2018-313005. 29921571

[17] Harris KM, Halpern CT, Biemer P, Liao D, Dean SC. Add health wave V documentation: sampling and mixed-mode survey design. Chapel Hill, NC: Carolina Population Center; 2019. http://www.cpc.unc.edu/projects/addhealth/documentation/guides.

[18] Resnick MD, Bearman PS, Blum RW, Protecting adolescents from harm. Findings from the national longitudinal study on adolescent health. JAMA. 1997;278(10):823–832. 10.1001/jama.278.10.823. 9293990

[19] Wu J, Xiong Y, Xia X, Can dementia risk be reduced by following the American Heart Association’s Life’s Simple 7? A systematic review and dose-response meta-analysis. Ageing Res Rev. 2023;83:101788. 10.1016/j.arr.2022.101788. 36371016

[20] Zheng Y, Huang T, Guasch-Ferre M, Estimation of life’s essential 8 score with incomplete data of individual metrics. Front Cardiovasc Med. 2023;10:1216693. 10.3389/fcvm.2023.1216693. 37564908 PMC10410141

[21] Radloff LS. The CES-D scale: a self-report depression scale for research in the general population. Appl Psychol Meas. 1977;1(3):385–401. 10.1177/014662167700100306.

[22] Arnett DK, Blumenthal RS, Albert MA, 2019 ACC/AHA guideline on the primary prevention of cardiovascular disease: a report of the American College of Cardiology/American Heart Association Task Force on Clinical Practice guidelines. Circulation. 2019;140(11):e596–e646. 10.1161/CIR.0000000000000678. 30879355 PMC7734661

[23] Powell-Wiley TM, Baumer Y, Baah FO, Social determinants of cardiovascular disease. Circ Res. 2022;130(5):782–799. 10.1161/CIRCRESAHA.121.319811. 35239404 PMC8893132

[24] Blackmore HL, Ozanne SE. Programming of cardiovascular disease across the life-course. J Mol Cell Cardiol. 2015;83:122–130. 10.1016/j.yjmcc.2014.12.006. 25510678

[25] Hardy R, Lawlor DA, Kuh D. A life course approach to cardiovascular aging. Future Cardiol. 2015;11(1):101–113. 10.2217/fca.14.67. 25606706 PMC4374150

[26] Gill D, Georgakis MK, Zuber V, Genetically predicted midlife blood pressure and coronary artery disease risk: Mendelian randomization analysis. J Am Heart Assoc. 2020;9(14):e016773. 10.1161/JAHA.120.016773. 32627641 PMC7660704

[27] Ghosh AK, Hughes AD, Francis D, Midlife blood pressure predicts future diastolic dysfunction independently of blood pressure. Heart. 2016;102(17):1380–1387. 10.1136/heartjnl-2015-308836. 27056972 PMC4998951

[28] Gray L, Lee IM, Sesso HD, Batty GD. Blood pressure in early adulthood, hypertension in middle age, and future cardiovascular disease mortality: HAHS (Harvard Alumni Health Study). J Am Coll Cardiol. 2011;58(23):2396–2403. 10.1016/j.jacc.2011.07.045. 22115646 PMC3253414

[29] Burford NG, Webster NA, Cruz-Topete D. Hypothalamic-pituitary-adrenal axis modulation of glucocorticoids in the cardiovascular system. Int J Mol Sci. 2017;18(10):2150. 10.3390/ijms18102150. 29035323 PMC5666832

[30] Seravalle G, Grassi G. Sympathetic nervous system and hypertension: new evidences. Auton Neurosci. 2022;238:102954. 10.1016/j.autneu.2022.102954. 35151003

[31] Choi JH, Kim MS. Homeostatic regulation of glucose metabolism by the central nervous system. Endocrinol Metab (Seoul). 2022;37(1):9–25. 10.3803/EnM.2021.1364. 35255598 PMC8901968

