# Supplementary material for: Biological Indicators of Cardiovascular Health by Foster Care History in Adults
Source: Am J Prev Med. Author manuscript; Available in PMC 2025 Nov 1. (PMC12577747; doi:10.1016/j.amepre.2025.108097)
Supplement: Supplementary Materials [file NIHMS2119116-supplement-Supplementary_Materials.pdf]

Biological indicators of cardiovascular health by foster care history in adults

Darlynn M. Rojo-Wissar, PhD, MPH[1,2,3], Sean R. Womack, PhD[1,2,3], Tomas Baka, MD, PhD[4], Adam P. Spira, PhD[5,6,7], Ryan D. Davidson, PhD[8], Eric S. Zhou, PhD[8], Candice A. Alfano, PhD[9], Chandra L. Jackson, PhD, MS[10,11], Michael A. Grandner, PhD[12] & Stephanie H. Parade, PhD[1,2,3]

[1]Department of Psychiatry and Human Behavior, Alpert Medical School of Brown University, Providence, RI, USA; [2]Bradley/Hasbro Children's Research Center, E.P. Bradley Hospital, East Providence, RI, USA; [3]Center of Biomedical Research Excellence for Stress, Trauma, and Resilience, The Miriam Hospital, Providence, RI, USA; [4]Institute of Pathophysiology, Faculty of Medicine, Comenius University, Bratislava, Slovakia; [5]Department of Mental Health, Johns Hopkins Bloomberg School of Public Health, Baltimore, MD; [6]Department of Psychiatry and Behavioral Sciences, Johns Hopkins School of Medicine, Baltimore, MD; [7]Center on Aging and Health, Johns Hopkins University, Baltimore, MD; [8]Boston Children's Hospital, Boston, Massachusetts, USA; Harvard Medical School, Boston, Massachusetts, USA; [9]Department of Psychology, University of Houston, Houston, TX, USA; [10]Epidemiology Branch, National Institute of Environmental Health Sciences, National Institutes of Health, Department of Health and Human Services, Research Triangle Park, NC, USA; [11]Intramural Program, National Institute on Minority Health and Health Disparities, National Institutes of Health, Department of Health and Human Services, Bethesda, MD, USA; [12]Department of Psychiatry, University of Arizona College of Medicine, Tucson, AZ, USA

American Journal of Preventive Medicine

Supplemental Materials

Biological indicators of cardiovascular health by foster care history in adults

Appendix 1: Biological Indicators of Cardiovascular Health

Appendix 2: Unweighted Prevalence (No.) of Complete Data and Weighted Prevalence of Missingness by Foster Care Placement History

Appendix 3: Biological Indicators of Cardiovascular Health by Negative Foster Care Experiences

Appendix 4: Discussion of Subgroup Analyses by Foster Care History and Contextual Factors

References

Appendix 1: Biological Indicators of Cardiovascular Health

Add Health field examiners measured participant height and weight, blood pressure, collected health history and medication information, and collected venous blood samples from participants, which were assayed for lipid and glucose concentrations. BMI was calculated using height and weight measurements. The variables of interest used from the blood samples were non-high-density lipoprotein cholesterol (non-HDL-C, mg/dL), Hemoglobin A1c (HbA1c, %), and fasting blood glucose (FBG, mg/dL) (which was used in scoring blood glucose health if HbA1c was missing). For blood pressure, the Add Health constructed variables for average systolic and diastolic blood pressure (mmHg) were used. Blood pressure was measured by trained field examiners using a factory-calibrated Microlife BP3MC1-PC-IB oscillometric monitor. Measurements were taken with participants seated and rested for 5 minutes, feet flat and legs uncrossed. Three readings were collected, each at least 30 seconds apart. During the measurement intervals, respondents were asked to raise their elbow to shoulder level and hold their forearm at a 90-degree angle for 5 seconds. Readings were recorded directly into a tablet, and device errors were addressed per manufacturer guidelines. The constructed variables for systolic and diastolic blood pressure represent the average of the second and third measurements. If either was missing, the other was used; if both were missing, the first measurement was used.

These data were used to create standardized scores for each biological indicator of CVH (BMI, blood pressure, blood glucose, blood lipids [non-HDL cholesterol]) on a scale of 0 to 100 using American Heart Association (AHA) criteria (**Appendix Table 1**). Higher scores indicate better CVH. In addition to computing individual scores for each biological indicator of CVH, scores on the 4 indicators were averaged to create an overall CVH score.

We also report mean values and 95% confidence intervals for the raw data that were used to create the AHA scores by foster care placement history in **Appendix Table 2.** Descriptive information on the raw data is reported overall, and in the context of reported medication use and disease status information that was used in the AHA scoring criteria.

Appendix Table 1. Scoring Criteria for the Four Biological Indicators of Cardiovascular Health, based on American Heart Association Guidelines

Factor	Scoring				
	**Points***	**BMI kg/m^2**			**Clinical Classification**
BMI	100	18.5-24.9			Optimal
	70	25.0-29.9			Overweight
	30	30.0-34.9			Obese
	15	35.0-39.9			Obese
	0	≥ 40			Obese
	**Points***	**Non-HDL Cholesterol (mg/dL)**			**Clinical Classification**
Blood Lipids	100	< 130			Optimal
	60	130-159	*If drug treated level (i.e. past 4-week antihyperlipidemic med use), subtract 20 points		Intermediate
	40	160-189			High
	20	190-219			High
	0	≥ 220			High
	**Points***	**Hemoglobin A1c (%)**		**Reported Diabetes History**	**Clinical Classification**
Blood Glucose	100	< 5.7 (OR Fasting Blood Glucose (FBG) <100 mg/dL)	AND	No	Optimal
	60	5.7-6.4 (OR FBG 100-125 mg/dL)	AND	No	Prediabetes
	40	6.5-6.9	AND	N/A	Diabetes
	30	7.0-7.9	AND	N/A	Diabetes
	20	8.0-8.9	AND	N/A	Diabetes
	10	9.0-9.9	AND	N/A	Diabetes
	0	≥ 10	AND	N/A	Diabetes
	*If no history of diabetes and drug treated level (i.e. past 4-week anti-diabetic med use) for A1c <6.5 OR FBG <125, subtract 20 points; Those with a history of diabetes for A1c <7.0 receive a score of 40. Five participants with FBG >125 were scored using the American Diabetes Association scoring calculator: https://professional.diabetes.org/glucose_calc				
	**Points***	**Systolic (mm Hg)**		**Diastolic (mm Hg)**	**Clinical Classification**
Blood Pressure	100	< 120	AND	< 80	Optimal
	75	120-129	AND	< 80	Elevated
	50	130-139	OR	80-89	Hypertension
	25	140-159	OR	90-99	Hypertension
	0	≥ 160	OR	≥ 100	Hypertension
	*If drug treated level, subtract 20 points				

Note. Scores range from 0 to 100, with scores of 80-100 (green) considered high, 50-79 (yellow) moderate, and 0-49 (red) considered low cardiovascular health.

Appendix Table 2. Raw Biomarkers of Cardiovascular Health Characteristics by Foster Care Placement History, weighted Means and 95% Confidence Intervals (CIs)

Characteristics	Foster Care Placement History					
	Yes (n= 113)			No (n=4,512)		
	Mean	95% CI		Mean	95% CI	
		LL	UL		LL	UL
BMI (including BMI<18.5)	31.86	29.87	33.85	30.77	30.30	31.24
BMI (excluding BMI<18.5)†	32.25	30.37	34.12	30.87	30.40	31.34
Past 4-week antihypertensive medication use (weighted %)	12.30%			12.66%		
Systolic Blood Pressure, overall	128.56	123.24	133.88	123.41	122.73	124.08
Systolic Blood Pressure (No past 4-week antihypertensive med use)	**128.60**	**122.82**	**134.38**	**122.08**	**121.44**	**122.73**
Diastolic Blood Pressure, overall	84.03	80.23	87.82	80.03	79.50	80.56
Diastolic Blood Pressure (No past 4-week antihypertensive med use)	**84.47**	**80.76**	**88.17**	**79.11**	**78.61**	**79.61**
Blood Sample: Fasting 8+ hours (weighted %)	54.26%			64.16%		
Past 4-week antihyperlipidemic medication use (weighted %)	8.81%			4.04%		
Non-HDL Cholesterol, overall	141.07	130.21	151.93	132.20	130.48	133.92
Non-HDL Cholesterol (No past 4-week antihyperlipidemic med use)	**145.26**	**134.64**	**155.89**	**131.95**	**130.13**	**133.78**
Self-reported History of Diabetes (weighted %)	10.06%			6.52%		
Past 4-week anti-diabetic medication use (weighted %)	3.72%			4.57%		
Had data on Hemoglobin A1C (weighted %)	92.22%			88.21%		
Hemoglobin A1C	5.32	5.21	5.43	5.37	5.33	5.41
Hemoglobin A1C (No past 4-week antihyperlipidemic med use or history of Diabetes)	5.24	5.16	5.32	5.24	5.21	5.27
Fasting Blood Glucose	90.82	86.85	94.79	94.05	92.19	95.91
Fasting Blood Glucose (No past 4-week antihyperlipidemic med use or history of Diabetes)	90.81	87.00	94.62	90.28	88.86	91.69

Note. †35 people (unweighted) have an underweight BMI, and these people were not included in the CVH scores used in the manuscript using AHA criteria (weighted %: FC history = 2.46%, no FC history = 0.78%). The unweighted correlation between Hemoglobin A1C and fasting blood glucose in this sample was 0.809, p = 0.000. LL = lower limit, UL = upper limit. Non-overlapping 95% CIs for the mean values by foster care placement history are bolded.

Appendix 2: Unweighted Prevalence (No.) of Complete Data and Weighted Prevalence of Missingness by Foster Care Placement History

Appendix Table 3. Unweighted Prevalence (No.) and Missingness (Unweighted No. [weighted %]) Characteristics of US Adults by Foster Care Placement History

Characteristics	Total Sample (n= 4,625)	Foster Care Placement History	
		Yes (n= 113)	No (n=4,512)
Sex (female)	2,813	73	2,740
Missing	0 (0%)	0 (0%)	0 (0%)
Race and Ethnicity			
NH White	2,824	58	2,766
NH Black	825	28	797
NH AIAN	18	0	18
NH Asian	206	7	199
Hispanic or Latino	546	10	536
Pacific Islander	22	0	22
NH Other Race	8	1	7
NH Multiracial	163	9	154
Missing	13 (.28%)	0 (0%)	13 (.28%)
Marital status			
Married	2,759	44	2,715
Widowed	14	0	14
Divorced	516	20	496
Separated	132	9	123
Never Married	1,196	40	1,156
Missing	8 (.18%)	0 (0%)	8 (.18%)
Poverty Index			
Low Income	570	28	542
Low Middle Income	298	15	283
Middle Income	950	18	932
High Income	2,381	38	2,343
Missing	426 (9.33%)	14 (12.36%)	412 (9.28%)
Depression			
Top 50% CES-D Scores	4,565	110	4,455
Missing	60 (1.6%)	3 (2.34%)	57 (1.58%)
Biological Indicators of CVH			
Overall CVH	3,855	95	3,760
Missing	770 (16.24%)	18 (13.26%)	752 (16.29%)
BMI	4,502	107	4,395
Missing	123 (2.55%)	6 (7.04%)	117 (2.47%)
Non-HDL Cholesterol	4,128	105	4,023
Missing	497 (10.26)	8 (5.88%)	489 (10.34%)
Blood Glucose	4,104	103	4,001
Missing	521 (10.83%)	10 (7.52%)	511 (10.89%)
Blood Pressure	4,432	108	4,324
Missing	193 (4.47%)	5 (.51%)	188 (4.54%)

Note. AIAN = American Indian or Alaska Native; CES-D = Center for Epidemiologic Studies Depression Scale; NH = Non-Hispanic/Latino. For age, 1 (.07%) participant was missing data, and they did not have a foster care placement history.

Appendix 3: Biological Indicators of Cardiovascular Health by Negative Foster Care Experiences

Among those with a FC placement history, 18 (11.9%, weighted to represent 37,297 adults) reported experiencing maltreatment in foster or adoptive care (26% (weighted) reported being adopted) and 40 (26.5%; weighted to represent 82,957 adults) reported experiencing more than one FC placement. There were no missing data on maltreatment history. One participant was missing data on the number of foster care placements.

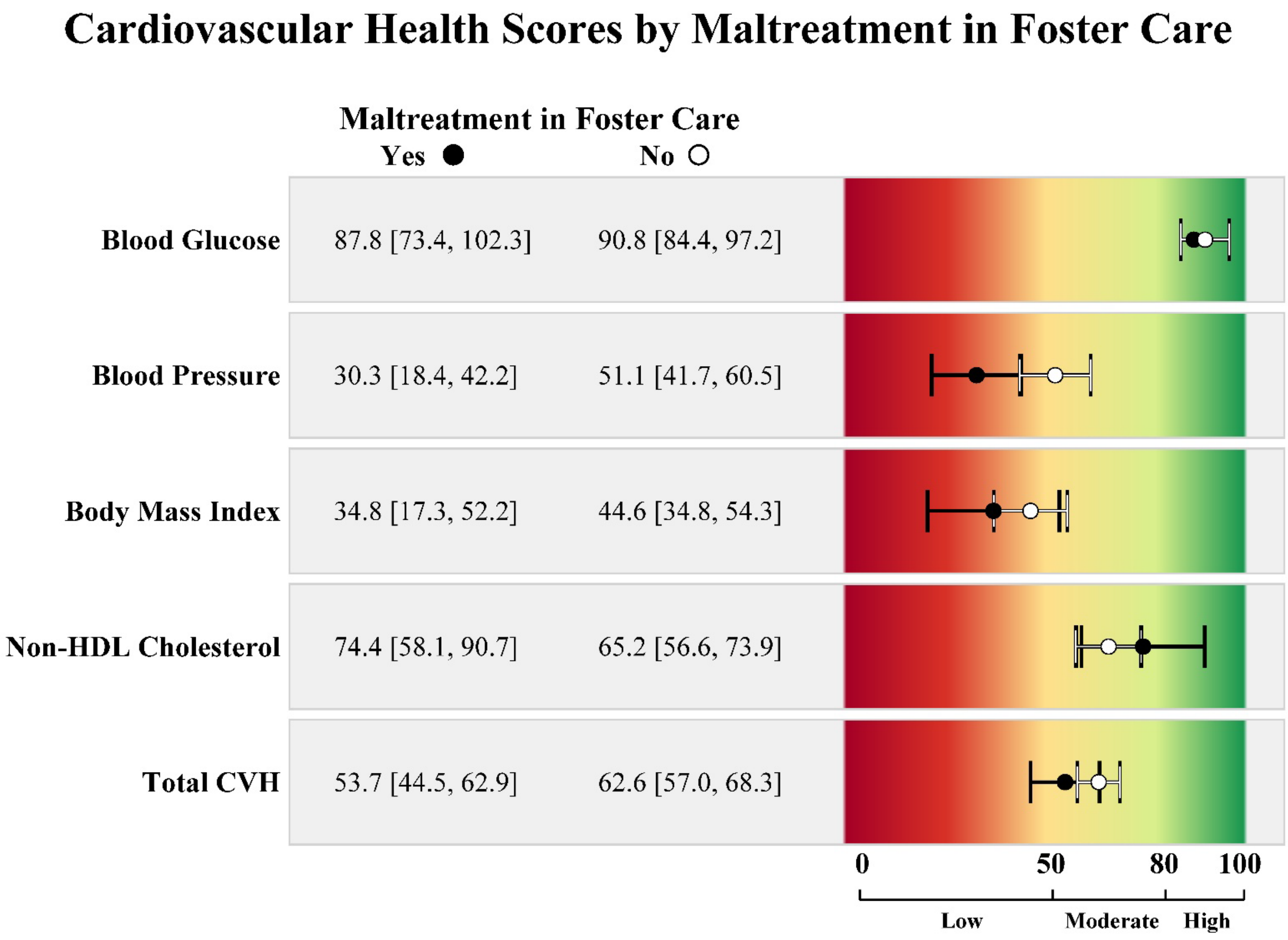

Appendix Figure 1. Biological Indicators of Cardiovascular Health (CVH) Scores by Maltreatment in Foster or Adoptive Care. Point estimates and 95% confidence intervals are in white for those with a maltreatment history and in black for those without. Mean scores are considered different by maltreatment history if 95% confidence intervals do not overlap and are indicated by asterisks.

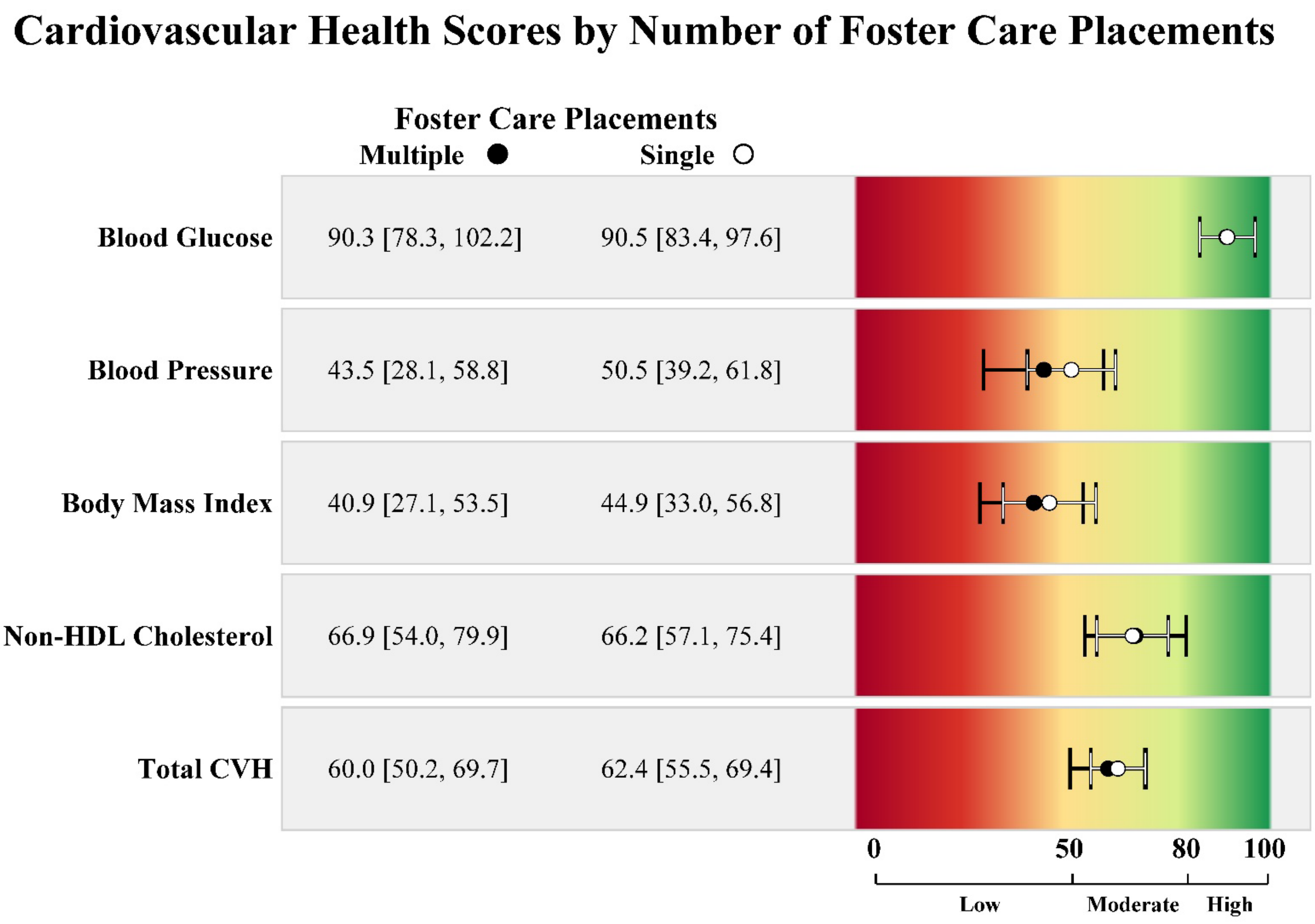

Appendix Figure 2. Biological Indicators of Cardiovascular Health (CVH) Scores by Number of Foster Care Placements. Point estimates and 95% confidence intervals are in white for those with a history of multiple foster care placements and in black for those with a single foster care placement history. Mean scores are considered different by number of placements if 95% confidence intervals do not overlap and are indicated by asterisks.

Appendix 4: Discussion of Subgroup Analyses by Foster Care History and Contextual Factors

Among those without a FC history, males had poorer overall CVH, blood pressure, and blood lipid scores relative to females, which is consistent with recent epidemiological research.[1] However, these sex differences were not observed among individuals with a FC placement history, due to females with a FC history exhibiting poorer scores on these CVH metrics compared to females without a FC history, but males showing no difference in scores on the CVH metrics by FC history status. Findings suggest that being female may no longer be protective for CVH in the face of a FC placement history. Sex differences in the association between childhood stressors and CVH are understudied,[2,3] but these findings are consistent with results from a recent meta-analysis that found that experiencing a FC placement in childhood was more strongly related to the development of CVD in adulthood among females.[4] Previous research also generally supports a stronger association between child maltreatment and risk for CVD in females.[5,6] Sex differences in adversity-related CVH risk may be attributable to differential exposure to certain maltreatment subtypes[6,7] and in the HPA-axis' response to stress,[8] but need further examination.

For race/ethnicity, overall CVH scores only differed by FC history among White adults, where those with a FC history had poorer overall CVH compared to those without. One possible explanation for a lack of differences in overall CVH by FC history among racially/ethnically minoritized groups is that their differential exposure to chronic stressors related to systemic racism and discrimination could obscure the effects of FC placement. When examining the individual biological indicators, interestingly, among Hispanic/Latino adults, those with a FC history had substantially poorer blood pressure but better blood lipid health (non-HDL cholesterol) compared to those without. Additional studies with large racially and ethnically diverse samples are needed to further our understanding of our findings and of group differences in health outcomes among adults with a history of FC.

Socioeconomic factors in adulthood, including higher income and being married, are associated with better CVH and lower risk for CVD.[9,10] Interestingly, in our sample, experiencing FC was only associated with poorer overall CVH and blood pressure scores for people in middle income and low-income brackets, and with poorer blood lipids and BMI scores only for people in middle income brackets. Unexpectedly, among high-income

adults, those with a FC history had better blood glucose scores compared to those without (although scores for both groups were in the high range). As mentioned previously in the context of lack of differences in overall blood glucose health scores by FC history, this could be explained by the strong homeostatic counter regulation of glucose levels by hormones like insulin. Poorer blood glucose health in those with a FC history may be more likely to appear in older age.

When examining marital status, those with a FC placement history had poorer overall CVH and BMI scores compared to those without, only among married adults. Overall CVH scores did not differ by marital status among adults with a FC history. Finally, among adults in the top, but not bottom, 50% of depressive symptom scores, individuals with a FC history exhibited poorer blood pressure health. Our findings suggest that experiencing FC may override previously observed protective effects of higher income and marital status and exacerbate existing CVH disparities for individuals with low income Further, elevated depressive symptoms may increase blood pressure health risk in this vulnerable population.

References

1. Lloyd-Jones DM, Ning H, Labarthe D, et al. Status of Cardiovascular Health in US Adults and Children Using the American Heart Association's New "Life's Essential 8" Metrics: Prevalence Estimates From the National Health and Nutrition Examination Survey (NHANES), 2013 Through 2018. *Circulation*. 2022;146(11):822-835. doi:10.1161/CIRCULATIONAHA.122.060911

2. Basu A, McLaughlin KA, Misra S, Koenen KC. Childhood Maltreatment and Health Impact: The Examples of Cardiovascular Disease and Type 2 Diabetes Mellitus in Adults. *Clinical Psychology: Science and Practice*. 2017;24(2):125-139. doi:10.1111/cpsp.12191

3. Suglia SF, Koenen KC, Boynton-Jarrett R, et al. Childhood and Adolescent Adversity and Cardiometabolic Outcomes: A Scientific Statement from the American Heart Association. *Circulation*. 2018;137(5):e15-e28. doi:10.1161/CIR.0000000000000536

4. David Batty G, Kivimäki M, Almquist YB, et al. Cardiovascular Disease Events in Adults with a History of State Care in Childhood: Pooling of Unpublished Results from 9 Cohort Studies. *medRxiv*. Published online January 27, 2024:2024.01.26.24301814. doi:10.1101/2024.01.26.24301814

5. Batten S V, Aslan M, Maciejewski PK, Mazure CM. *Childhood Maltreatment as a Risk Factor for Adult Cardiovascular Disease and Depression*. Vol 65.; 2004.

6. Soares ALG, Hammerton G, Howe LD, Rich-Edwards J, Halligan S, Fraser A. Sex differences in the association between childhood maltreatment and cardiovascular disease in the UK Biobank. *Heart*. 2020;106(17):1310-1316. doi:10.1136/heartjnl-2019-316320

7. McLaughlin KA, Koenen KC, Hill ED, et al. Trauma exposure and posttraumatic stress disorder in a national sample of adolescents. *J Am Acad Child Adolesc Psychiatry*. 2013;52(8). doi:10.1016/j.jaac.2013.05.011

8. Heck AL, Handa RJ. Sex differences in the hypothalamic–pituitary–adrenal axis' response to stress: an important role for gonadal hormones. *Neuropsychopharmacology*. 2019;44(1):45-58. doi:10.1038/s41386-018-0167-9

9. Johnson-Lawrence V, Kaplan G, Galea S. Socioeconomic mobility in adulthood and cardiovascular disease mortality. *Ann Epidemiol*. 2013;23(4):167-171. doi:10.1016/j.annepidem.2013.02.004

10. Manfredini R, De Giorgi A, Tiseo R, et al. Marital Status, Cardiovascular Diseases, and Cardiovascular Risk Factors: A Review of the Evidence. *J Womens Health*. 2017;26(6):624-632. doi:10.1089/jwh.2016.6103
